# Negative symptoms and associated factors in older people with schizophrenia

**DOI:** 10.1192/j.eurpsy.2023.781

**Published:** 2023-07-19

**Authors:** M. Karoui, I. kammoun, R. Kammoun, H. Nefzi, F. Ellouze

**Affiliations:** 1 Tunis, Faculté de médecine de Tunis, Tunis, Tunisia; 2 Faculté de médecine de Tunis, Tunis

## Abstract

**Introduction:**

The evolution of schizophrenia with age remains poorly studied. The prevalence of negative symptoms in elderly people with schizophrenia is even less described in the literature.

**Objectives:**

to evaluate the prevalence of remission of negative symptoms in the elderly and to study the sociodemographic and clinical variables associated with this remission.

**Methods:**

The sample consisted of 83 subjects aged 55 years and over, followed at the psychiatry department "G" of the Razi hospital in Tunis and suffering from schizophrenia according to the DSM5 criteria. Global remission was defined as a score below 4 on the seven negative symptom items of the PANSS. A questionnaire was administered to each patient to collect epidemiological and anamnestic data.

**Results:**

59% of the sample showed remission of global negative symptoms. 84% and 60% were in remission on the emotional and cognitive subscales, respectively. The existence of remission was correlated with lower PANSS global score, more preserved cognitive functioning, later age of onset, more family and social support, and the absence of a concomitant somatic illness.

**Conclusions:**

This study showed that measures to optimize treatment of positive symptoms and cognitive functioning may have an impact on negative symptoms. Similarly, quality of social network in later life impacts the level of negative symptoms.

**Disclosure of Interest:**

None Declared

